# *In vivo* and *in vitro* protein imaging in thermophilic archaea by exploiting a novel protein tag

**DOI:** 10.1371/journal.pone.0185791

**Published:** 2017-10-03

**Authors:** Valeria Visone, Wenyuan Han, Giuseppe Perugino, Giovanni del Monaco, Qunxin She, Mosè Rossi, Anna Valenti, Maria Ciaramella

**Affiliations:** 1 Institute of Biosciences and Bioresources, National Research Council of Italy, Napoli, Italy; 2 Department of Biology, University of Copenhagen, Copenhagen, Denmark; Istituto di Genetica Molecolare, ITALY

## Abstract

Protein imaging, allowing a wide variety of biological studies both *in vitro* and *in vivo*, is of great importance in modern biology. Protein and peptide tags fused to proteins of interest provide the opportunity to elucidate protein location and functions, detect protein-protein interactions, and measure protein activity and kinetics in living cells. Whereas several tags are suitable for protein imaging in mesophilic organisms, the application of this approach to microorganisms living at high temperature has lagged behind. Archaea provide an excellent and unique model for understanding basic cell biology mechanisms. Here, we present the development of a toolkit for protein imaging in the hyperthermophilic archaeon *Sulfolobus islandicus*. The system relies on a thermostable protein tag (H5) constructed by engineering the alkylguanine-DNA-alkyl-transferase protein of *Sulfolobus solfataricus*, which can be covalently labeled using a wide range of small molecules. As a suitable host, we constructed, by CRISPR-based genome-editing technology, a *S*. *islandicus* mutant strain deleted for the alkylguanine-DNA-alkyl-transferase gene (*Δogt*). Introduction of a plasmid-borne H5 gene in this strain led to production of a functional H5 protein, which was successfully labeled with appropriate fluorescent molecules and visualized in cell extracts as well as in *Δogt* live cells. H5 was fused to reverse gyrase, a peculiar thermophile-specific DNA topoisomerase endowed with positive supercoiling activity, and allowed visualization of the enzyme in living cells. To the best of our knowledge, this is the first report of *in vivo* imaging of any protein of a thermophilic archaeon, filling an important gap in available tools for cell biology studies in these organisms.

## Introduction

Detection and analysis of proteins in their cellular context and under their physiological conditions is crucial for understanding their function. Over the last few decades, great progress has been obtained toward this task by exploiting fluorescent protein tags, such as the Green Fluorescent Protein (GFP) and its derivatives, and these proteins can be fused to proteins of interest enabling a wide variety of biological studies [1,2,3].

**Fig 1:**
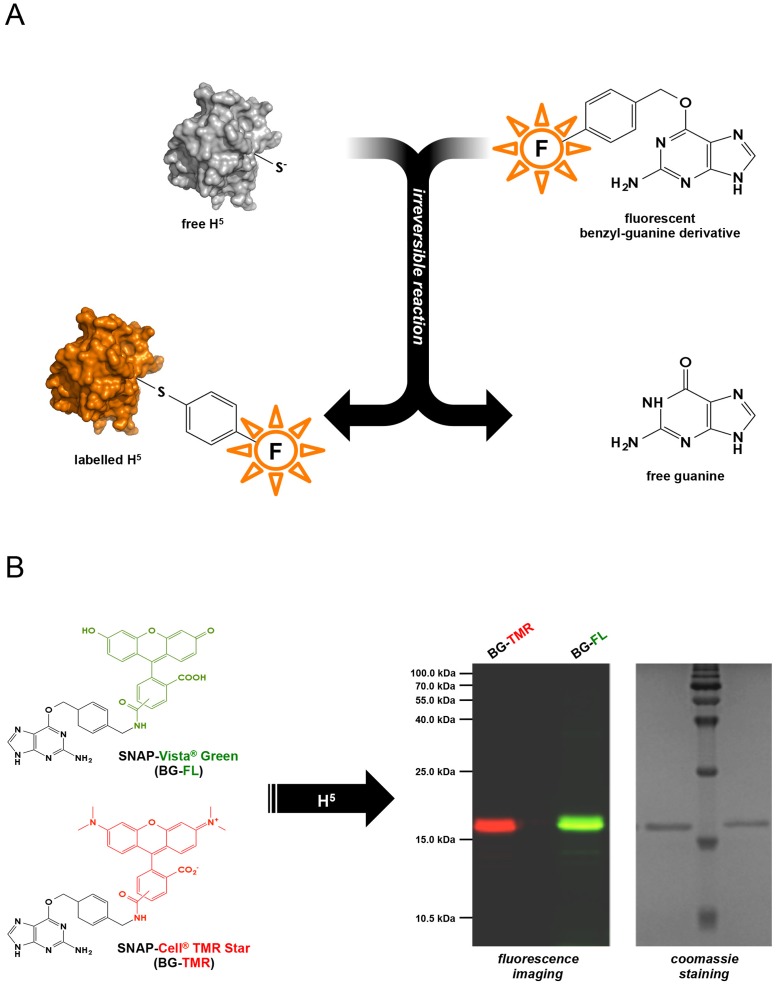
H5 labeling. A. Scheme of the reaction leading to irreversible labeling of H5 with a fluorescent derivative of benzyl-guanine. B. SDS-PAGE of purified H5 labeled with two different benzyl-guanine derivatives.

As an alternative to GFP-based systems, other protein and peptide tags have been recently introduced [2]. One of the most promising is the so-called SNAP-tag, proposed by K. Johnsson and colleagues [4,5]. This approach exploits the unique properties of a protein involved in repair of alkylation damage in DNA, the O^6^-alkylguanine-DNA alkyl-transferase (AGT or OGT). Upon reaction of this protein with O^6^-benzylguanine (BG), a small molecule acting as irreversible inhibitor of the enzyme, a covalent bond between the benzyl group and a specific cysteine residue in the protein active site is formed. If BG is conjugated with a suitable chemical group (such as a fluorophore, biotin, and so on), this latter is transferred to the protein molecule, thus resulting in covalent labeling of AGT and AGT-containing chimeric proteins (Fig 1A) [6]. Although this approach relies on the addition of an external substrate, which cells may not be permeable to, it holds several advantages as compared with GFPs: it is extremely specific, highly versatile, and offers the possibility to label proteins with virtually unlimited chemical groups [6].

Because both GFP and SNAP-tag are mesophilic proteins, *in vitro* they can only function under mild reaction conditions, and their use for *in vivo* imaging has been essentially restricted to organisms living in the range of mesophilic temperatures. Although thermotolerant GFPs variants have been recently produced [7,8], the application of protein tags to thermophilic and hyperthermophilic microorganisms (both bacteria and archaea) has lagged behind.

We have previously obtained an engineered version of the OGT protein from the hyperthermophilic and acidophilic archaeon *Sulfolobus solfataricus* [9,10]. This modified protein, named H5, was obtained by mutation of five aminoacid residues in the protein helix-turn-helix domain, thus impairing the DNA binding activity; like the wild-type OGT, H5 can be effectively labeled with fluorophores or other chemical groups conjugated with a benzyl-guanine (Fig 1B), and is thus suitable as a candidate protein tag for thermophilic organisms.

As a typical *S*. *solfataricus* protein, H5 displayed a strong stability under harsh conditions, including high temperature, extremes of pH, ionic strength, presence of organic solvents and digestion with proteases [10]. The H5 protein was fused to the *S*. *solfataricus* β-glycosidase, giving rise to a chimeric protein which was correctly expressed, folded, functional and stable in both *Escherichia coli* and the thermophilic bacterium *Thermus thermophilus*, and could be imaged in living cells as well as in cell-free protein extracts [10]. Thus, H5 behaved like a thermostable version of the commercial SNAP-tag protein, which is widely used for studies in mesophilic organisms [4,11].

So far no protein tag has been reported for hyperthermophilic archaea. The cell biology of these organisms is of particular interest, not only for their peculiar lifestyle, but also because their cell machineries devoted to DNA replication and repair, and gene transcription share common evolutionary origin with those of eukaryotes. Therefore, these organisms provide good model systems to study the most basic mechanisms of genome-related processes in the life branch of Eukarya and Archaea.

One of the most fascinating and mysterious proteins of hyperthermophilic organisms is reverse gyrase (RG). It is a unique DNA topoisomerase that introduces positive supercoils into DNA molecules (for reviews, see [12,13,14]) and is exclusively found in organisms living above 60°C [15,16], thus suggesting that the enzyme plays a role in adaptation to high temperatures. Accordingly, positive supercoiling is predicted to protect DNA from denaturation at the growth temperatures of hyperthermophiles. A number of studies support a role for RG in DNA protection, repair and response to DNA damage: it acts as a DNA renaturase, promoting annealing of complementary single-stranded DNA circles [17]; binds to single stranded breaks on DNA and prevents DNA thermal denaturation at the DNA breaks [18]; is degraded after treatment of *S*. *solfataricus* cells with alkylating agents, in concomitance with degradation of genomic DNA [19]; interacts with and inhibits the translesion DNA polymerase PolY and the single-strand DNA binding protein, SSB [20,21]; is recruited to DNA after ultraviolet irradiation [22]. In addition, RG is able to resolve *in vitro* Holliday junctions following both ATP-dependent and ATP-independent mechanisms [23,24].

Despite biochemical and structural data, the function of this enzyme is still under debate. Genetic studies in different archaeal species gave contradictory results: a RG knock out strain of *Thermococcus kodakaraensis* was viable, but showed slower growth at higher temperatures, as compared with the wild type [25]. In contrast, deletion of the RG gene in *Pyrococcus furiosus* was lethal at temperatures higher than 95°C [26]; finally, the two RG encoding genes of the crenarchaeon *Sulfolobus islandicus* were both essential for growth at any temperature [27]. Thus, whereas RG is likely involved in thermotolerance and fundamental DNA-related process at high temperature, its absence seems to have different degrees of severity in different species. The reasons for these discrepancies are currently unknown and many gaps are still present in our understanding of the function of this peculiar topoisomerase.

Here, we present the development of a thermostable protein tag suitable for protein visualization in the hyperthermophilic crenarcheaon *S*. *islandicus*, one of the most useful archaeal models for genetic studies [28]. To this aim, we constructed, by a CRISPR-based genome-editing method [29], a *S*. *islandicus* mutant strain deleted for the *ogt* gene (*Δogt*). This strain was transformed with an autonomously replicating plasmid expressing the H5 protein. The protein was successfully expressed and could be efficiently labeled using specific fluorescent substrates in *S*. *islandicus* living cells. In order to demonstrate the utility of this tag, we fused H5 to the TopR1 RG from *S*. *solfataricus*. The chimeric protein was correctly folded when expressed in *E*. *coli* and retained both the alkyl-transferase and positive supercoiling activities; moreover, the fusion protein was successfully expressed and functional in *S*. *islandicus Δogt* cells transformed with a suitable expression vector. The potential applications of this technology for cell biology studies in thermophilic organisms will be discussed.

## Results and discussion

### Construction of a *S*. *islandicus ogt-KO* strain

In a proof-of-concept work, we have previously shown that the H5 protein can be successfully used as a protein tag in the thermophilic bacterium *T*. *Thermophilus* [10]; this organism was a convenient model to demonstrate the usefulness of our tag because it is genetically tractable and lacks endogenous DNA alkyl-transferase activity [30].

**Fig 2:**
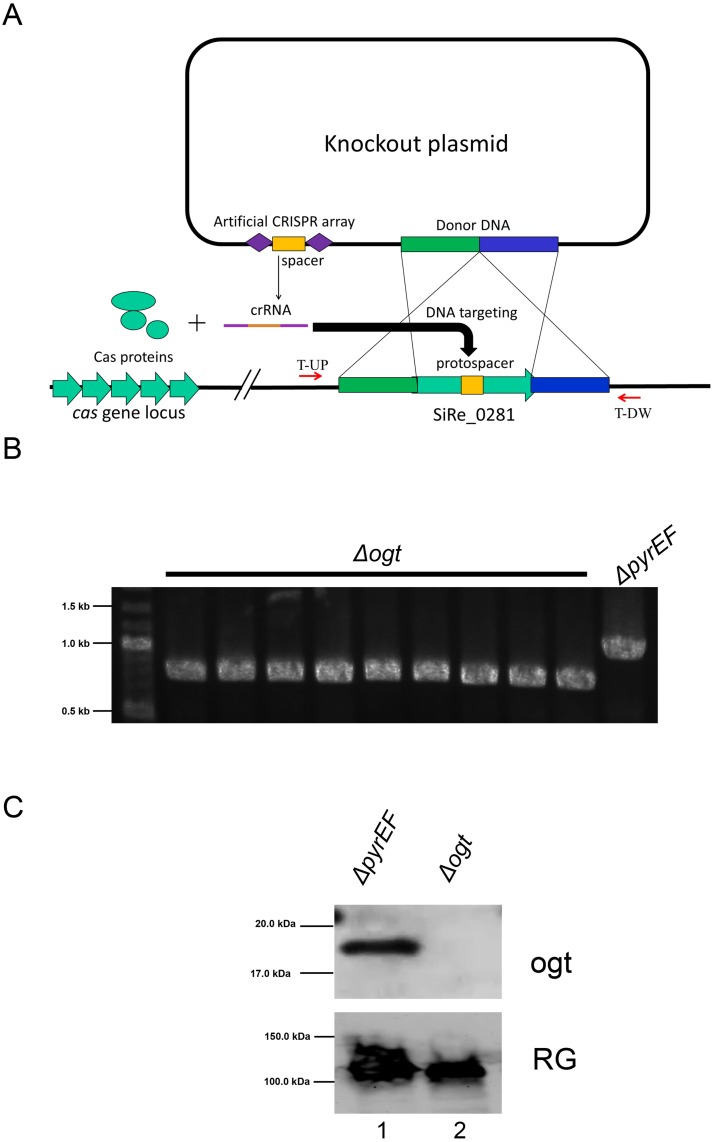
Construction and analysis of a *S*. *islandicus Δogt* strain. A. Scheme of the construction of the *S*. *islandicus ΔpyrEF ogt-KO* derivative strain (*Δogt*). B. PCR test of nine *Δogt* strains and *ΔpyrEF* as a control. The primers (T-UP and T-DW) are indicated in (A). C. Western blot analysis of total cell extracts (400 μg/lane) prepared from cultures of *ΔpyrEF* (lane 1) and *Δogt* (lane 2) strains. The filter was probed with the antibody against the *S*. *solfataricus* OGT protein [9]; as a control for protein loading, the same filter was stripped and probed against the *S*. *solfataricus* TopR1 protein [21].

We sought to extend the application of our system to hyperthermophilic archaea, for which no protein tag is available. Although these organisms are prokaryotes, they use regulatory elements and mechanisms distinct from bacteria. Genetic manipulation has recently became possible only for a limited number of hyperthermophilic archaeal species, thus we needed to identify an organism suitable for our purpose, i. e., transformable and selectable, lacking endogenous DNA alkyl-transferase activity and permeable to the H5 substrate. We choose *S*. *islandicus*, for which vectors, transformation and gene knock out systems are available. Preliminary tests showed that *S*. *islandicus* cells are permeable to the H5 substrate (data not shown; see below). However, the *S*. *islandicus* genome contains an ORF (SiRe_0281) potentially coding for an OGT ortholog, which shares 99% similarity with the *S*. *solfataricus ogt* gene (data not shown). Since the activity of this protein might interfere with our assay, a prerequisite for the application of the H5 tag was the construction of a *S*. *islandicus* mutant strain in which the *ogt* gene was deleted by using the CRISPR-based genome-editing method which has recently been developed for this species [29]. The knockout plasmid (pKO-*ogt*) was constructed as described in Materials and Methods. The plasmid contains a donor DNA fragment carrying an in-frame deletion in the *ogt* gene and an artificial mini-CRISPR array with a spacer matching a protospacer in the *ogt* gene sequence, and importantly, the protospacer is absent from the *ogt* deletion allele (Fig 2A). Upon transformation of the plasmid into *S*. *islandicus* E233S1, two genetic events would occur in the transformants: (a) recombination between the donor DNA and the wild-type *ogt* gene locus would yield the desired *ogt* mutant allele on the chromosome, and (b) crRNA generated from the expression of the plasmid-borne mini-CRISPR array would form ribonucleoprotein complexes with Cas proteins encoded by the endogenous CRISPR-Cas system and guide them to mediate self-targeting on the chromosomes containing the wild-type *ogt* gene (Fig 2A). Since *ogt* deletion mutants lack the protospacer, they are not targeted by the CRISPR activity, whereas the wild-type chromosome is targeted for DNA degradation. Therefore, colonies of pKO-*ogt* transformants grown on uracil-free nutrient plates should be the designed *ogt* deletion strain. Indeed, characterization of nine colonies by PCR amplification of the *ogt* deletion allele from the candidate mutants using the T-UP and T-DW primer set (Table 1) and agarose gel electrophoresis of the resulting PCR products revealed that all nine colonies only contained the *ogt* deletion allele (Fig 2B). Furthermore, DNA sequencing of the PCR products revealed that the designed mutation was obtained, which retained a short truncated peptide of 6 amino acids in the *ogt* deletion allele. The strain, hereafter called *Δogt*, was viable at 75°C and the absence of the OGT protein was confirmed by western blot of total cell extracts, which showed a band reacting with the antibody directed against the *S*. *solfataricus* OGT protein [9] in the *ΔpyrEF* strain, but not in *Δogt* (Fig 2C).

**Table 1:** Oligonucleotides used for the construction of the *S*. *islandicus Δogt* strain.

Name	Sequence
**KOSiRe_0281spF**	5’-aagCTTGGCTATATAACTGTTGCTAAGGACGATAAGGGATTTA-3’
**KOSiRe_0281spR**	5’-agcTAAATCCCTTATCGTCCTTAGCAACAGTTATATAGCCAAG-3’
**KOSiRe_0281Lf**	5’-ctttgcatgcCCGCGTTGCAAGAATCGGGC-3’
**KOSiRe_0281Lr**	5’-CCACATCATTCCCCATACACTAGCACAAGTATTAAT-3’
**KOSiRe_0281Rf**	5’-GCTAGTGTATGGGGAATGATGTGGAAAAATTTAACAG-3’
**KOSiRe_0281Rr**	5’-gtttctcgagCCATCCCTTGTTTCTCTACG-3’
**KOSiRe_0281T-UP**	5’-CTAAGACAGTGGAAGTTTGGC-3’
**KOSiRe_0281T-DW**	5’-CCACGTCTTGGTTGTCCAGTC-3’

### Heterologous expression of SsOGT-H5 in *S*. *islandicus* and *in vivo* labeling

**Fig 3:**
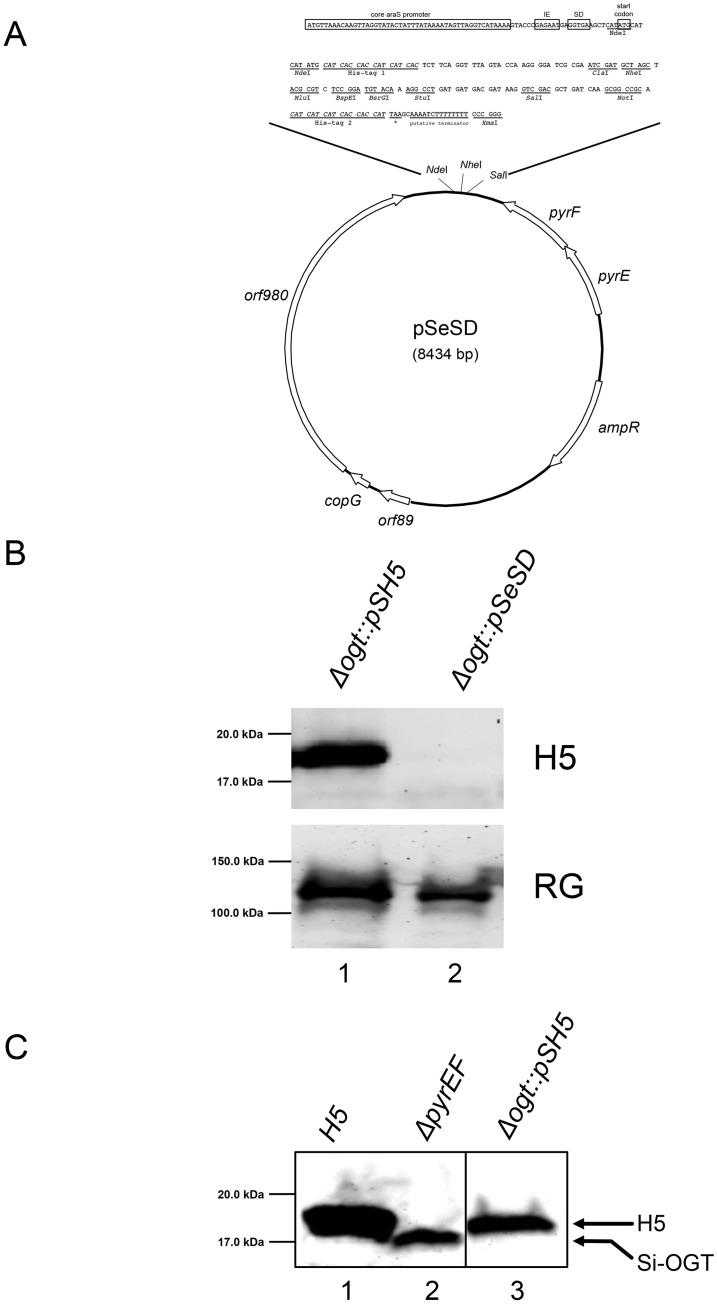
Heterologous expression of H5 in the *S*. *islandicus Δogt* strain. A. The pSeSD plasmid used to transform *S*. *islandicus* [32]. The *H5* and *H5-TopR1* genes were inserted in the *NdeI-SalI* and *NdeI-NheI* restriction sites, respectively. B. Western blot analysis of cell extracts (400 μg/lane) prepared from cultures of the *Δogt* strain transformed with either pSH5 (lane 1) or the pSeSD empty vector (lane 2). The same filter was stripped and probed with antibodies against the indicated proteins. C. Western blot analysis using the anti-OGT antibody [9]. Lane 1: purified H5 protein (400 ng); lane 2: *S*. *islandicus ΔpyrEF* cell extracts (400 μg); lane 3: *Δogt* transformed with pSH5 cell extracts (300 μg). The arrows indicate the migration of endogenous *S*. *islandicus* OGT (Si-OGT) and heterologous H5.

Having obtained a suitable host, the next step was to introduce the H5 protein in *S*. *islandicus* cells. To this aim, we constructed an expression vector suitable to transform the *Δogt* strain based on uracil selection. Strains carrying the *ΔpyrEF* mutation are unable to produce uracil that is necessary for the growth in minimal liquid medium (SCV medium); transformants can be selected for by complementation, provided that a functional *pyr*EF cassette is present in the plasmid. Complementation-based strategies are a convenient choice for *Sulfolobus* species, also because of the limited success in the use of antibiotics as selective markers in hyperthermophilic Archaea [31]. To construct the expression vector, the *SsOgt-H5* gene was cloned in the pSeSD plasmid (Fig 3A) [32], which carries a synthetic arabinose-inducible promoter that confers high levels of protein expression. The resultant recombinant plasmid was called pSH5. The pSH5 plasmid was introduced in the *Δogt* strain by electroporation, and transformants were selected by growth in a liquid selective medium (SCV, see Materials and methods) at 75°C; after 5 days, transformed cultures reached ∼0.7/0.8 OD_600_. The presence of the H5 protein in transformants was confirmed by western blot (Fig 3B). The H5 protein was expressed from the plasmid-borne gene at about 0.2 ng μg^-1^ of total protein extract, which was comparable to the level of the endogenous OGT protein in the parental *ΔpyrEF* strain (Fig 3C).

**Fig 4:**
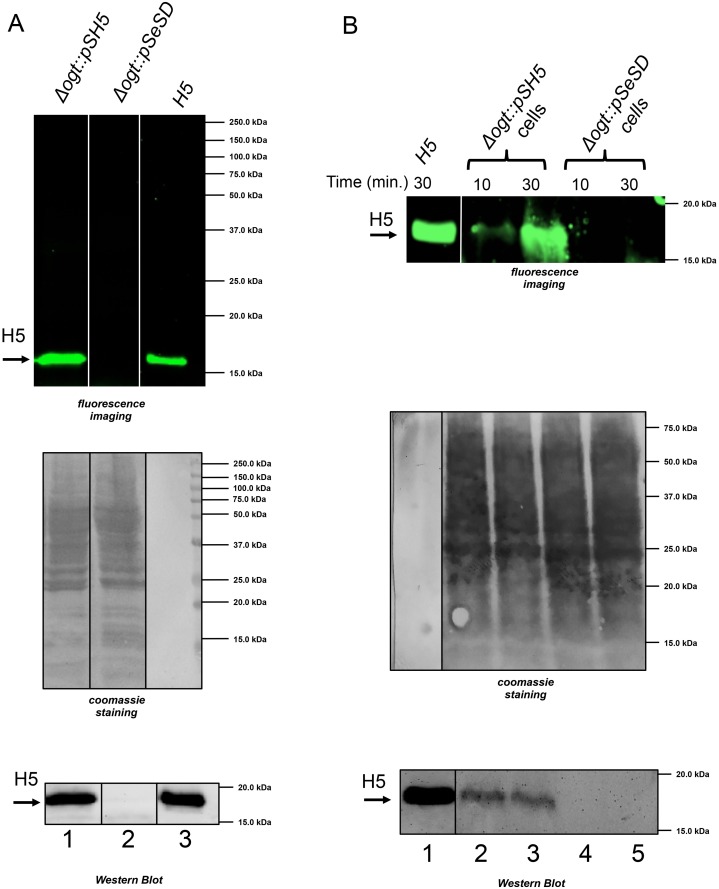
Activity of the H5 protein in *S*. *islandicus*. A. Cell free extracts (200 μg) prepared from cultures of the *Δogt* strain transformed with pSH5 (lane 1) or the pSeSD empty vector (lane2); lane 3 contains 100 ng of the H5 protein purified from *E*. *coli*. Samples were incubated with the BG-FL substrate (2.5 μM) for 30 min. at 70°C and loaded on SDS-PAGE; the gel was exposed for fluorescence imaging analysis, blotted and stained with Coomassie blue. Finally, the filter was probed with the anti-OGT antibody (bottom panel). B. Permeability of *S*. *islandicus* to the BG-FL substrate. Whole transformed cells were incubated in SCV medium in the presence of 3.0 μM of BG-FL, at 70°C for times as indicated. Lane 1 contains 100 ng of purified H5 protein; lanes 2 and 3 contain *Δogt* cells transformed with pSH5; lanes 4 and 5 contain *Δogt* cells transformed with pSeSD. The gel was exposed for fluorescence imaging analysis, blotted and stained with Coomassie blue. Finally, the filter was probed with the anti-OGT antibody (bottom panel).

We next wanted to test whether the H5 protein was functional in transformants. To this aim, we applied a fluorescent assay we previously developed, which utilizes a fluorescent derivative of the BG substrate (BG-FL) [9,10]. BG-FL was incubated with cell free extracts prepared from cultures of the *Δogt* strain transformed with either pSH5 or the pSeSD empty vector. After incubation at 70°C for 30 min., samples were denatured and loaded on SDS-PAGE for fluorescence imaging analysis. A fluorescent band of the expected molecular weight was observed in extracts from cells transformed with pSH5, but not pSeSD, thus confirming that the H5 protein is correctly expressed and proficient for fluorescent labeling in the heterologous host (Fig 4A).

In order to test the permeability of *S*. *islandicus* cells to the BG-FL substrate, intact *Δogt* cells transformed with either pSH5 or pSeSD were incubated in the presence of BG-FL at 70°C for different times; after reaction, cells were washed, denatured and loaded on SDS-PAGE for fluorescence imaging analysis. As shown in Fig 4B, the results were very similar to those obtained with cells extracts (compare Fig 4A and 4B), with a strong fluorescent band seen in *Δogt* cells transformed with pSH5, but not the empty plasmid. This result suggests that *S*. *islandicus* cells are permeable to the BG-FL substrate.

**Fig 5:**
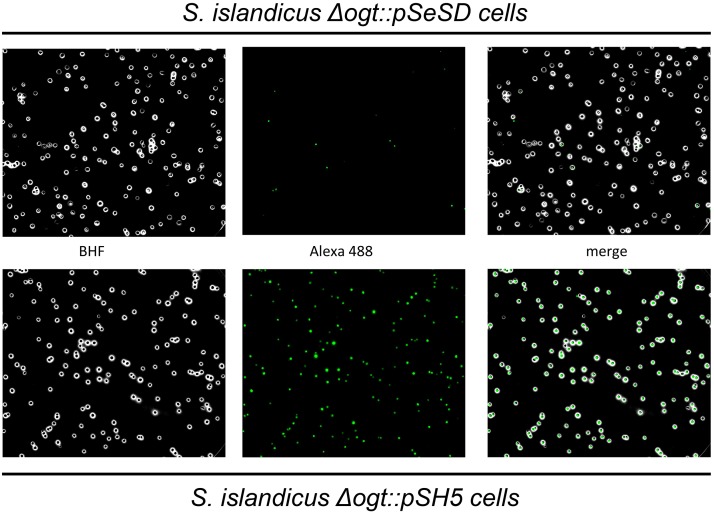
Fluorescence microscopy. *S*. *islandicus Δogt* cells transformed with the empty vector (top) or with the pSH5 plasmid (bottom) were incubated with BG-FL (3 μM) and then analysed at fluorescence microscopy. Images show bright field (BHF), AlexaFluor488 (green) and merged images.

This result was confirmed by fluorescence microscopy (Fig 5). Intact *Δogt* cells transformed with the empty pSeSD incubated with BG-FL and washed as described in the Material and Methods section, showed little no fluorescent signals; in contrast, strong fluorescent signals appeared in cells transformed with the pSH5 plasmid, indicating that *S*. *islandicus* cells are permeable to the BG-FL substrate and that the observed labeling is specific for the H5 protein. Quantitative analysis showed that more than 80% of cells transformed with pSH5, but only about 5% of those transformed with pSeSD showed fluorescent signals, suggesting that the plasmid is stably retained during growth and the H5 protein is correctly expressed, folded and active in the vast majority of transformed cells. These results showed that our thermostable H5 tag is suitable for protein localization and analysis in this hyperthermophilic archaeon.

### Application of the H5 tag to detection of RG in *S*. *islandicus*

**Fig 6:**
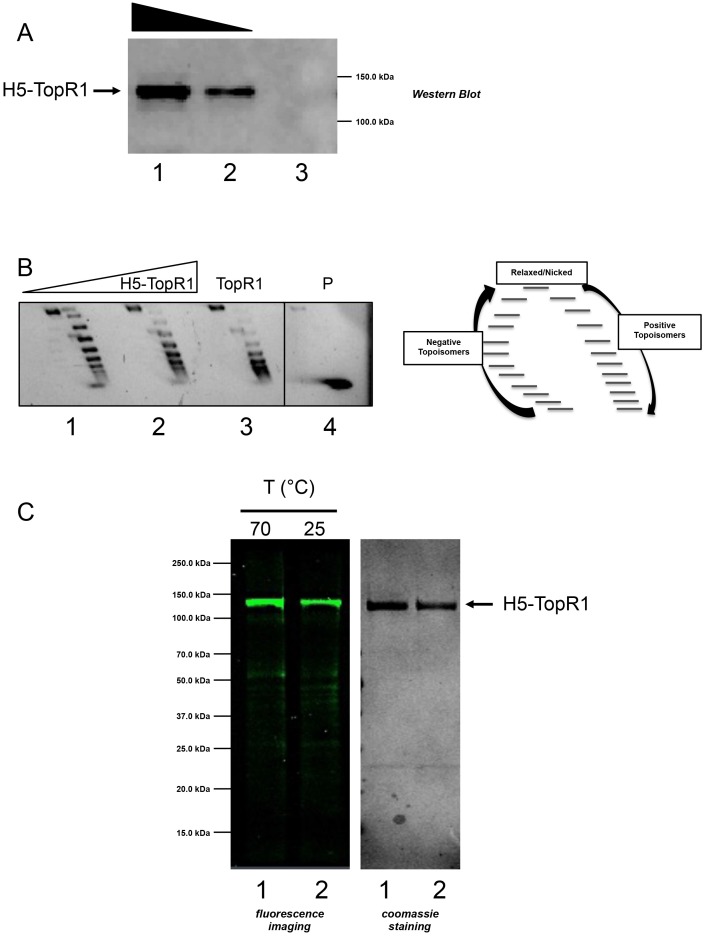
Analysis of the H5-TopR1 chimeric protein expressed in *E*. *coli*. A. Western blot analysis of cell extracts prepared from *E*. *coli* ABLE C cultures transformed with the pQE-H5-TopR1 plasmid (lane 1 and 2, 50 and 25 μg respectively) or with the pQE empty vector (lane 3, 50 μg). B. Positive supercoiling assay. Left panel: negatively supercoiled DNA was incubated with 1 or 5 μg of H5-TopR1 protein extract for 10 min. at 85°C (lane 1 and 2, respectively); lane 3: negatively supercoiled DNA incubated with purified RG (60 nM) for 10 min. at 85°C; lane 4: not-incubated negatively supercoiled DNA. Right panel: diagram of 2D gel showing the migration of plasmid topoisomers. C. Labelling of H5-TopR1 purified from *E*. *coli* cells. Purified H5-TopR1 fusion protein (1.0 μM) was incubated with the BG-FL substrate (5 μM) for 30 min. at 70°C (lane 1) or for 1 h at 25°C (lane 2). After SDS-PAGE, the gel was exposed for the fluorescence imaging analysis (left) and stained with Coomassie blue (right).

In order to validate the suitability of H5 as a thermostable tag for protein imaging, we sought to visualize RG in *S*. *islandicus* cells. To this aim, the *S*. *solfataricus topR1* gene was fused downstream to and in frame with the *H5* gene, obtaining the chimeric *H5-topR1* gene. To be sure that the presence of the H5 tag at the N-terminal end of the enzyme does not affect RG activity, we first sought to test the functionality of the fusion protein. To this aim, the chimeric gene was cloned in the pQE vector and introduced in *E*. *coli* ABLE C strain. The fusion protein was successfully expressed in *E*. *coli*, as shown by western blot analysis (Fig 6A). The ability of the fusion protein to introduce positive supercoils into DNA molecules was assayed in total protein extracts as previously reported [20,33]. As showed in Fig 6B, protein extracts containing the fusion protein exhibit positive supercoiling activity, indicating that the presence of the H5 tag does not impair the enzyme activity.

Purification of the fusion protein by affinity chromatography through a nickel column was performed, exploiting the presence of a Hisx6-tag at the N-terminus and the thermostability of both RG and H5 to perform a thermoprecipitation step (data not shown). To test whether the fusion protein might be labeled by the fluorescent substrate, we applied the same alkyl-transferase assay described above [9]. SDS-PAGE showed the presence of a fluorescent band of the expected molecular weight (Fig 6C), indicating that the H5 portion is functional in the fusion protein. As previously shown for H5 [10], the chimeric protein could be labeled at both 25 and 70°C, although, as expected, more efficiently at the latter temperature (Fig 6C).

**Fig 7:**
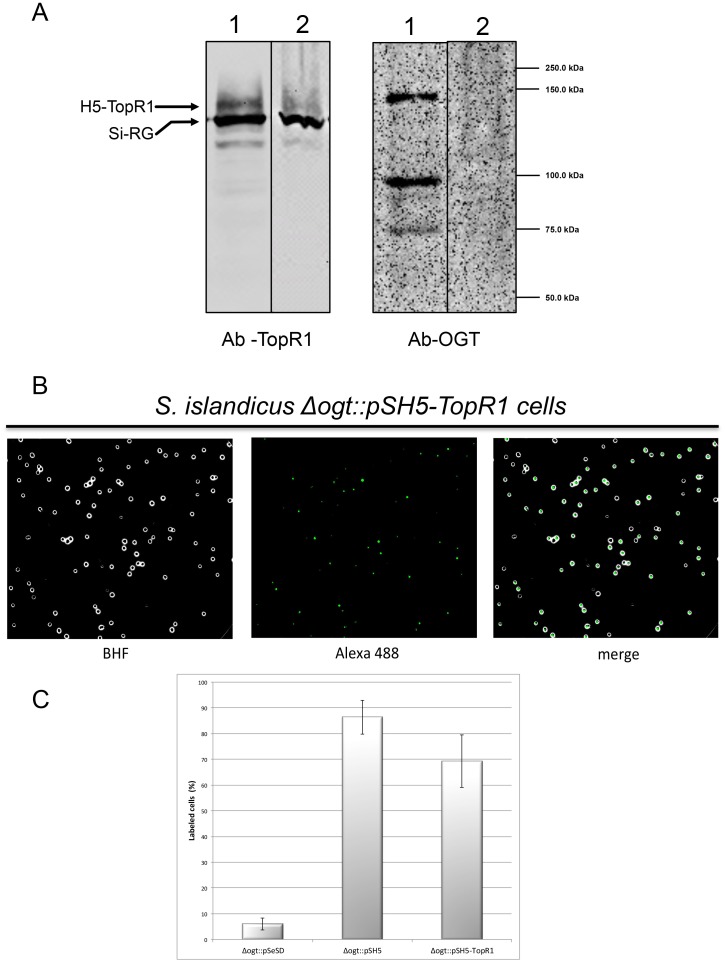
H5-TopR1 heterologous expression in *S*. *islandicus Δogt* strain. A. Western blot analysis of protein cell extracts (100 μg/lane) prepared from cell cultures transformed with pSH5-TopR1 (lane 1) or the pSeSD empty vector (lane 2). The same filter was stripped and probed with antibodies against the indicated proteins. The arrows indicate the migration of endogenous *S*. *islandicus* RG (Si-RG) and heterologous H5-TopR1. B. *In vivo* protein labeling: fluorescence microscopy of *S*. *islandicus Δogt* cells transformed with pSH5-TopR1 plasmid incubated with BG-FL (3 μM). Images show bright field (BHF), AlexaFluor488 (green) and merged images. C. Quantitative analysis of labeled cells in the *Δogt* strain transformed with the indicated plasmids. The percentage of labeled cells was calculated considering about 100 cells per sample in three independent experiments. Bars represent standard deviation.

In order to visualize RG in *S*. *islandicus*, cells, the H5-TopR1 coding sequence was cloned in the pSeSD plasmid and the resultant vector, called pSH5-TopR1, was used to transform the *S*. *islandicus Δogt* mutant. Transformants were able to grow at 75°C showing no apparent growth defect (data not shown). The presence of the H5-TopR1 protein in transformants was confirmed by western blot (Fig 7A): an antibody directed against *S*. *solfataricus* TopR1 recognized one band in the *Δogt* strain transformed with pSeSD, corresponding to endogenous *S*. *islandicus* protein (which shares 91% sequence identity with TopR1). In the same strain transformed with pSH5-TopR1 the antibody recognized two bands, corresponding to endogenous RG and H5-TopR1, respectively; assuming that the affinity of the antibody for the two proteins is similar, we estimated that the fusion is expressed at approximately the same levels as the endogenous RG. Interestingly, the anti-OGT antibody revealed the presence of smaller bands in extracts of cells transformed with pSH5-TopR1 (Fig 7A, right). Since these shorter fragments were not evidenced by the anti-TopR1 antibody, we conclude that these fragments contain H5 and N-terminal portions of TopR1, and could be due to either degradation or premature termination of the chimeric protein (see also below). Degradation and/or premature termination has been reported for both endogenous and heterologously expressed *S*. *solfataricus* RG [19,34].

The H5-TopR1 protein was visualized *in vivo* by fluorescence microscopy analysis of intact *S*. *islandicus* cells (Fig 7B). As shown above for cells expressing H5 alone, we observed specific fluorescent signals in cells transformed with pSH5-TopR1, thus showing that the H5-TopR1 protein is proficient for labeling in living cells. Quantitative analysis showed that about 70% of fusion expressing cells could be labeled with the fluorescent substrate, a lower fraction as compared with cells expressing H5 alone (Fig 7C). This result might be due to plasmid loss, uneven level of expression of the H5-TopR1 protein in single cells or degradation of the chimeric protein. This latter hypothesis is supported by western blot data (see Fig 7A).

These data show that our thermostable H5 tag is suitable for protein localization and analysis in hyperthermophilic archaea and provide the opportunity for further *in vivo* and *in vitro* studies on the biological roles of RG (positive supercoiling, repair, protection), making thus possible to study the enzyme under its physiological conditions, without the need to remove the tag.

## Conclusions

We developed a novel protein tagging system for a hyperthermophilic archaeon. The system, based on the H5 protein tag and a purposely obtained *S*. *islandicus* host strain, offers broad flexibility for both *in vivo* imaging and biochemical applications with a single tag that binds rapidly, covalently, and specifically small molecules. We have validated the usefulness of the system by tagging the thermophile-specific RG protein, showing that the H5 tag allows visualization of the enzyme in *S*. *islandicus* live cells without interfering with its positive supercoiling activity.

Although we have previously reported the use of the H5 protein tag in a thermophilic bacterium, the present work is novel in several respects. Indeed, to the best of our knowledge, this is the first time a protein tag is reported to work in live cells of any hyperthermophilic archaeon, and the first time not only for reverse gyrase, but any protein is imaged *in vivo* in these organisms. Despite our previous successful experience with the use of H5 in *T*. *thermophilus*, the same success was not granted in *S*. *islandicus* because of the completely different genetic background. Besides adaptation of our previously developed protocols and construction of new plasmids, it was also necessary to construct a *S*. *islandicus* strain deleted for the endogenous alkyltransferase gene, which was possible thanks to the recently developed CRISPR-Cas9 genome editing method for this species [29]. This novel strain might be also useful to study the *in vivo* function and regulation of the archaeal OGT protein, which is only partially understood [9].

Our data pave the way for future investigations on the function of RG, which, despite mechanistic and structural studies, remains enigmatic. In addition, the H5 tag might be used as a thermostable version of the SNAP-tag protein in other (hyper)thermophilic archaea, for which genetic tools are available. Applications include detection and sub-cellular localization of proteins and protein interactions. Different fluorescent ligands could be used to label different proteins, including in pulse-chase analysis, and follow their movements and fate in the cell in real time.

## Materials and methods

### Reagents

All chemicals were purchased from Sigma-Aldrich; SNAP-Vista^®^ Green substrate (referred to as BG-FL) was from New England Biolabs (Ipswich, MA). Synthetic oligonucleotides were from Primm (Milan, Italy) and listed in Tables 1 and 2; *Pfu* DNA polymerase was from Stratagene (La Jolla, CA), as well as *Escherichia coli* ABLE C strain.

**Table 2:** Oligonucleotides used for the construction of the pSH5, pQE-ogtH5-topR1 and pSeSD-H5-topR1 plasmids.

Name	Sequence
**H5-up NdeI**	5’-GCGATATCCATATGCTGGTGTATGGATTGTATAAAAG-3’
**H5-dw SalI**	5’-GTACGTCGACTTCTGGAATTTTGACTCCTTCC-3’
**H5-fwd**	5’-AAATAGGCGTATCACGAGGCCC-3
**H5-rev**	5’-GCATCAGAGCTCATTTCTGGAATTTTGACTCCTTCC-3’
**pQE_upstream-fwd**	5'-GTTGAGATCCAGTTCGATGTAACCC-3’
**H5_Nde-rev**	5'-GGTGATGGTGAGATCCTCTCATATGAGTTAATTTCTCCTCTTTAATG-3’
**H5_Nde-fwd**	5'-CATTAAAGAGGAGAAATTAACTCATATGAGAGGATCTCACCATCACC-3’

### Archaeal and bacterial strains used in this work

The hyperthermophilic archaeal genetic host *S*. *islandicus* E233S1 carrying Δ*pyrEF* mutation [35] was derived from *S*. *islandicus* REY15A [36]. The strain was used for generation of Δ*pyrEF*::*Δogt* (*Δogt*) by a CRISPR-based genome editing technology as described previously [29]. The strain was confirmed by PCR analysis using the primers T-UP and T-DW listed in Table 1.

*Δogt* cells carrying the pSeSD-based expression plasmids were employed for producing recombinant proteins. The strains were grown at 75°C in a medium containing basic salts supplemented with 0.2% sucrose, 0.2% Casamino Acids, plus a vitamin mixture (SCV). Cells were transferred to ACV in which D-arabinose was substituted for the other sugar (e.g., sucrose in SCV) to elevate recombinant proteins production.

*E*. *coli* ABLE C was used for H5-TopR1 purification. Cells were cultured at 22°C in Luria-Bertani (LB) medium, and ampicillin was added to 100 μg/ml.

### DNA constructs

The plasmid for generation of the *Δogt* strain was constructed as described previously [29]. Specifically, primers KOSiRe_0281spF and KOSiRe_0281spR (Table 1) were annealed, yielding a DNA fragment containing the spacer that is complementary to a protospacer selected from the coding sequence of the *S*. *islandicus ogt* gene [37]. The resulting DNA fragment was inserted into the spacer-cloning vector pSe-RP [38], giving pAC-*ogt*, an artificial mini-CRISPR plasmid containing a single spacer array matching the *ogt* protospacer. Then, donor DNA of the *ogt* deletion allele (retaining a coding sequence of 6 amino acids) was generated by the splicing overlap extension PCR [39] using primers listed in Table 1 and inserted into the pAC-ogt plasmid, giving the knockout plasmid (pKO-*ogt*). The knockout plasmid was transformed into the genetic host *S*. *islandicus* E233S1 [35] via electroporation, and transformants obtained on uracil-free nutrient plates should be mutants carrying the *ogt* deletion allele since cells carrying the wild-type *ogt* gene were selectively killed from self targeting of the endogenous CRISPR-Cas systems (Fig 2A). Plasmids present in the mutants were then removed by the *pyrEF* counter-selection using 5-fluorooratic acid, and the resulting *ogt* mutants were verified by DNA sequencing.

In order to construct the pSH5 plasmid, the *S*. *solfataricus ogtH5* gene was PCR-amplified from the pQE-*ogtH5* plasmid [9] using H5-up NdeI and H5-dw SalI primers (Table 2). This allowed us to insert *ogtH5* gene in the multi-cloning site of pSeSD plasmid for the heterologous expression of H5 in *S*. *islandicus*. To obtain the H5-TopR1 fusion protein, the *ogtH5* gene was PCR-amplified from the pQE-*ogtH5* construct [9] using H5-fwd and H5-rev oligonucleotides (Table 2), which possess a *SacI* site. The amplified gene was inserted in the pQE-*topR1* construct [40], upstream and in frame with the *S*. *solfataricus topR1* gene, leading to the pQE-*ogtH5*-*topR1* plasmid for expression of the H5-TopR1 fusion protein in *E*. *coli*. The pSeSD-*H5-topR1* plasmid was obtained by multiple rounds of PCR amplification: the pQE_upstream-fwd/H5_Nde-rev oligonucleotides pairs (Table 2) were first used to introduce *NdeI* site upstream *H5* sequence. In a second round of PCR, the H5_Nde-fwd/H5-rev oligonucleotides pairs (Table 2) were used to obtain a DNA fragment that overlaps the product of the first PCR round. Finally, the former two DNA fragments were fused to each other by using the external pQE_upstream-fwd/H5-rev oligonucleotides pairs. The obtained DNA fragment was ligated in the *NdeI*/*NheI* sites of the pSeSD vector. For all the obtained constructs, regions encoding the cloned genes were verified by DNA sequencing (Primm, Milan, Italy).

### Protein expression and purification

Expression plasmids were introduced into *S*. *islandicus* Δ*pyrEF* or Δ*ogt* via electroporation; transformants were grown in minimal liquid medium (SCV) and tested for expression of recombinant proteins. Cell free extracts were prepared from cultures until the cell density reached ∼0.7/0.8 OD_600_. Cells were harvested by centrifugation and resuspended in PBS 1× buffer (phosphate buffer 20 mM, NaCl 150 mM, pH 7.3). After disruption with sonication, cell debris was removed by centrifugation (10,000 x *g* for 30 min. at 4°C) and the supernatant was used to test the presence of the recombinant proteins.

H5-TopR1 was expressed in *E*. *coli* ABLE C and purified by Hisx6-tag FPLC; then, to remove *E*. *coli* contaminants, positive fractions eluted from the affinity column were incubated for 10 min at 80°C, and centrifuged for 30 min. at 30,000 x *g*. The soluble fraction was dialysed against PBS 1× buffer (phosphate buffer 20 mM, NaCl 150 mM, pH 7.3) and aliquots stored at −20°C. To assess the purity of the protein samples and determine their concentrations, SDS-PAGE and Bio-Rad protein assay were performed, respectively.

### Western blot

Total and fractionated protein extracts were prepared as previously described [22]. Samples were run in 4–12% gradient gels in 1x Tris-Glycine SDS Running buffer (25 mM Tris, 192 mM glycine and 0.1% SDS, pH 8.3). After electrophoresis, proteins were transferred onto PVDF filters (Bio-Rad) using the Trans-Blot^®^ Turbo^™^ Blotting System (Bio-Rad). Reagents used were: polyclonal antibodies raised in rabbit against *S*. *solfataricus* OGT [9] and TopR1 reverse gyrase [21] as primary antibodies; the goat anti-rabbit IgG-HRP (Pierce) as secondary antibody and the Amersham Biosciences ECL Plus kit. Filters were incubated, washed and developed according to manufacturer’s instructions. Chemiluminescent bands were revealed using a VersaDoc apparatus (Bio-Rad) and the QuantityOne software (Bio-Rad) was used for quantitation.

### Positive supercoiling assay

Positive supercoiling assay was performed using plasmid pBluescript (Qiagen) as a substrate, as previously reported [20,33]. Briefly, different amounts of protein extracts (1–5 μg) prepared from *E*. *coli* H5-TopR1-expressing cultures or purified TopR1 (60 nM) were incubated, at 85°C for 10 min. in a total volume of 20 μL, with 300 ng of the substrate in 1× RG buffer (35 mM Tris-HCl, pH 7.0, 0.1 mM Na_2_EDTA, 30 mM MgCl_2_, 2.0 mM DTT, 1 mM ATP). Plasmid topoisomers were separated by 2D agarose gel electrophoresis with ethidium bromide (0.01 μg/ml) in the second dimension. After electrophoresis, gel was stained with ethidium bromide (1 μg ml^-1^), and analysed under UV light with a VersaDoc 4000^™^ system (Bio-Rad, Hercules, CA, USA).

### *In vitro* labeling

Labeling of the H5 protein and the H5-TopR1 fusion by the BG-FL substrate was obtained as previously described [9,41,42]. For H5 expressed in *S*. *islandicus Δogt*, 200 μg of cell free extracts prepared from transformed cultures were incubated with BG-FL (2.5 μM) in 1× Fluo Reaction Buffer (50 mM phosphate, 0.1 M NaCl, 1.0 mM DTT, pH 6.5) at 70°C for 30 min. Reactions were stopped by denaturation and samples were subjected to SDS-PAGE, followed by fluorescence imaging analysis using a VersaDoc 4000^™^ system (Bio-Rad) by applying as excitation/emission parameters a blue LED bandpass filter.

The activity of the H5-TopR1 fusion protein purified from *E*. *coli* was measured by incubating 1.0 μM of protein with 5 μM of the BG-FL substrate in 1× Fluo Reaction Buffer at the indicated temperatures and time spans. Samples were analysed as described above.

To test the permeability of *S*. *islandicus* cells to BG-FL, *Δogt* cells transformed with either pSH5 or pSeSD were grown at 75°C in SCV selective medium until the stationary phase (OD_600_ ∼ 0.8). Cell pellets from 1 mL cultures were resuspended in 0.1 mL of SCV medium in the presence of 3.0 μM BG-FL and incubated at 70°C for 10 or 30 min. After the reaction, cells were washed twice with 1.0 mL of SCV medium, then denatured for 10 min. at 110°C in O’Farrell 1× buffer supplemented with EDTA 10 mM, and finally subjected to SDS-PAGE and fluorescence imaging analysis as described above.

### Protein imaging in *S*. *islandicus* cells

For *in vivo* imaging, *Δogt* cells transformed with the pSeSD, pSH5 or pSH5-TopR1 plasmids were grown at 75°C in SCV selective medium until OD_600_ ∼ 0.8. Cell pellets from 1.0 mL cultures were resuspended in 0.1 mL of SCV medium in the presence of 3.0 μM of BG-FL and incubated at 70°C for 30 min. After the reaction, cells were washed four times in phosphate-saline buffer (PBS) and then spotted on poly-L-lysine coated slides for microscopy analysis. A Nicon Eclipse Ti microscope attached to a Cool-Snap HQ CCD camera (Roper Scientific) was used to capture the images; excitation and emission wavelengths used suitable for Alexa Fluor 488 dye were λ_ex_ = 490 nm; λ_em_ = 525 nm, respectively. The images were processed using the ImageJ software.

## Supporting information

S1 File Original SDS PAGE gels and western blot filters. (A). Fluorescence imaging of fig. 1B. (B) Coomassie staining of Fig1B. (C). Fluorescence imaging of fig. 4A (*top*). (D) Fluorescence imaging of fig 4B (top). (E) Fluorescence imaging of fig. 6C. (F) Coomassie staining of Fig. 6C. (G) Western blot Fig 2C (top). (H) Western blot Fig 2C (down) (I) Western blot Fig 3B (top) (J) Western blot of Fig 3B (down) (K) Western blot Fig 3C. (L) Coomassie staining of filter of Fig. 4A (middle) (M) Western blot of Fig 4 A (down) (N) Coomassie staining of filter of Fig 4B (middle) (O) Western blot of Fig. 4B (down) (P) Western blot of Fig 6A (Q) Western blot of Fig. 7A (left) (R) Western blot of Fig.7A (right). (S) Ethidium bromide agarose gel of Fig. 2B. (T) 2D agarose gel after Ethidium bromide staining of Fig 6B.
(PPTX)

## References

[1] Chalfie M, Tu Y, Euskirchen G, Ward WW, Prasher DC. Green fluorescent protein as a marker for gene expression. Science 1994; 5148:802–805. 10.1126/science.8303295 8303295

[2] Johnsson N, Johnsson K. A fusion of disciplines: chemical approaches to exploit fusion proteins for functional genomics. Chembiochem. 2003; 4(9): 803–10. doi: 10.1002/cbic.200200603 12964152 10.1002/cbic.200200603

[3] Schneider AF, Hackenberger CP. Fluorescent labelling in living cells. Curr Opin Biotechnol. 2017; 48:61–68. doi: 10.1016/j.copbio.2017.03.012 28395178 10.1016/j.copbio.2017.03.012

[4] Juillerat A, Gronemeyer T, Keppler A, Gendreizig S, Pick H, Vogel H, et al Directed evolution of *O*^*6*^-alkylguanine-DNA alkyltransferase for efficient labeling of fusion proteins with small molecules *in vivo*. Chem Biol. 2003; 10(4): 313–7. 12725859 10.1016/s1074-5521(03)00068-1

[5] Gautier A, Juillerat A, Heinis C, Corrêa IR Jr, Kindermann M, Beaufils F, et al An engineered protein tag for multiprotein labeling in living cells. Chem Biol. 2008; 15(2): 128–36. doi: 10.1016/j.chembiol.2008.01.007 18291317 10.1016/j.chembiol.2008.01.007

[6] Hinner MJ, Johnsson K. How to obtain labeled proteins and what to do with them. Curr Opin Biotechnol. 2010; 21(6): 766–76. doi: 10.1016/j.copbio.2010.09.011 21030243 10.1016/j.copbio.2010.09.011

[7] Aliye N, Fabbretti A, Lupidi G, Tsekoa T, Spurio R. Engineering color variants of green fluorescent protein (GFP) for thermostability, pH-sensitivity, and improved folding kinetics. Appl Microbiol Biotechnol. 2015; 99(3): 1205–16. doi: 10.1007/s00253-014-5975-1 25112226 10.1007/s00253-014-5975-1

[8] Cava F, de Pedro MA, Blas-Galindo E, Waldo GS, Westblade LF, et al Expression and use of superfolder green fluorescent protein at high temperatures *in vivo*: a tool to study extreme thermophile biology. Environ Microbiol. 2008; 10(3): 605–13. doi: 10.1111/j.1462-2920.2007.01482.x 18190515 10.1111/j.1462-2920.2007.01482.x

[9] Perugino G, Vettone A, Illiano G, Valenti A, Ferrara MC, Rossi M, et al Activity and regulation of archaeal DNA alkyltransferase: conserved protein involved in repair of DNA alkylation damage. J Biol Chem. 2012; 287(6): 4222–31. doi: 10.1074/jbc.M111.308320 22167184 10.1074/jbc.M111.308320 PMC3281690

[10] Vettone A, Serpe M, Hidalgo A, Berenguer J, del Monaco G, Valenti A, et al A novel thermostable protein-tag: optimization of the *Sulfolobus solfataricus* DNA- alkyl-transferase by protein engineering. Extremophiles 2016; 20(1): 1–13. doi: 10.1007/s00792-015-0791-9 26499124 10.1007/s00792-015-0791-9

[11] Gronemeyer T, Chidley C, Juillerat A, Heinis C, Johnsson K. Directed evolution of *O*^*6*^-alkylguanine-DNA alkyltransferase for applications in protein labeling. Protein Eng Des Sel. 2006; 19(7): 309–16. doi: 10.1093/protein/gzl014 16638797 10.1093/protein/gzl014

[12] Perugino G, Valenti A, D'amaro A, Rossi M, Ciaramella M. Reverse gyrase and genome stability in hyperthermophilic organisms. Biochem Soc Trans. 2009; 37(Pt 1): 69–73. doi: 10.1042/BST0370069 19143604 10.1042/BST0370069

[13] Lulchev P, Klostermeier D. Reverse gyrase—recent advances and current mechanistic understanding of positive DNA supercoiling. Nucleic Acids Res. 2014; 42(13): 8200–13. doi: 10.1093/nar/gku589 25013168 10.1093/nar/gku589 PMC4117796

[14] Visone V, Vettone A, Serpe M, Valenti A, Perugino G, Rossi M, et al Chromatin structure and dynamics in hot environments: architectural proteins and DNA topoisomerases of thermophilic archaea. Int J Mol Sci. 2014; 15(9): 17162–87. doi: 10.3390/ijms150917162 25257534 10.3390/ijms150917162 PMC4200833

[15] Forterre P. A hot story from comparative genomics: reverse gyrase is the only hyperthermophile-specific protein. Trends Genet. 2002; 18(5): 236–7. 12047940 10.1016/s0168-9525(02)02650-1

[16] Brochier-Armanet C, Forterre P. Widespread distribution of archaeal reverse gyrase in thermophilic bacteria suggests a complex history of vertical inheritance and lateral gene transfers. Archaea 2007; 2(2): 83–93. 17350929 10.1155/2006/582916 PMC2686386

[17] Hsieh TS, Plank JL. Reverse gyrase functions as a DNA renaturase: annealing of complementary single-stranded circles and positive supercoiling of a bubble substrate. J Biol Chem. 2006; 281(9): 5640–7. doi: 10.1074/jbc.M513252200 16407212 10.1074/jbc.M513252200

[18] Han W, Feng X, She Q. Reverse Gyrase Functions in Genome Integrity Maintenance by Protecting DNA Breaks *In Vivo*. Int J Mol Sci. 2017; 18(7). pii: E1340. doi: 10.3390/ijms18071340 28640207 10.3390/ijms18071340 PMC5535833

[19] Valenti A, Napoli A, Ferrara MC, Nadal M, Rossi M, Ciaramella M. Selective degradation of reverse gyrase and DNA fragmentation induced by alkylating agent in the archaeon *Sulfolobus solfataricus*. Nucleic Acids Res. 2006; 34(7): 2098–108. doi: 10.1093/nar/gkl115 16617150 10.1093/nar/gkl115 PMC1440885

[20] Napoli A, Valenti A, Salerno V, Nadal M, Garnier F, Rossi M, et al Functional interaction of reverse gyrase with single-strand binding protein of the archaeon *Sulfolobus*. Nucleic Acids Res. 2005; 33(2): 564–76. doi: 10.1093/nar/gki202 15673717 10.1093/nar/gki202 PMC548347

[21] Valenti A, Perugino G, Nohmi T, Rossi M, Ciaramella M. Inhibition of translesion DNA polymerase by archaeal reverse gyrase. Nucleic Acids Res. 2009; 37(13): 4287–95. doi: 10.1093/nar/gkp386 19443439 10.1093/nar/gkp386 PMC2715243

[22] Napoli A, Valenti A, Salerno V, Nadal M, Garnier F, Rossi M, et al Reverse gyrase recruitment to DNA after UV light irradiation in *Sulfolobus solfataricus*. J. Biol. Chem. 2004; 279: 33192–33198. doi: 10.1074/jbc.M402619200 15190074 10.1074/jbc.M402619200

[23] Valenti A, Perugino G, Varriale A, D'Auria S, Rossi M, Ciaramella M. The archaeal topoisomerase reverse gyrase is a helix-destabilizing protein that unwinds four-way DNA junctions. J Biol Chem. 2010; 285(47): 36532–41. doi: 10.1074/jbc.M110.169029 20851892 10.1074/jbc.M110.169029 PMC2978581

[24] Jamroze A, Perugino G, Valenti A, Rashid N, Rossi M, Akhtar M, et al The reverse gyrase from *Pyrobaculum calidifontis*, a novel extremely thermophilic DNA topoisomerase endowed with DNA unwinding and annealing activities. J Biol Chem. 2014; 289(6): 3231–43. doi: 10.1074/jbc.M113.517649 24347172 10.1074/jbc.M113.517649 PMC3916527

[25] Atomi H, Matsumi R, Imanaka T. Reverse gyrase is not a prerequisite for hyperthermophilic life. J Bacteriol. 2004; 186(14): 4829–33. doi: 10.1128/JB.186.14.4829-4833.2004 15231817 10.1128/JB.186.14.4829-4833.2004 PMC438624

[26] Lipscomb GL, Hahn EM, Crowley AT, Adams MWW. Reverse gyrase is essential for microbial growth at 95°C. Extremophiles 2017; 21(3): 603–608. doi: 10.1007/s00792-017-0929-z 28331998 10.1007/s00792-017-0929-z

[27] Zhang C, Tian B, Li S, Ao X, Dalgaard K, Gökce S, Liang Y, et al Genetic manipulation in *Sulfolobus islandicus* and functional analysis of DNA repair genes. Biochem Soc Trans. 2013; 41(1): 405–10. doi: 10.1042/BST20120285 23356319 10.1042/BST20120285

[28] Peng N, Han W, Li Y, Liang Y, She Q. Genetic technologies for extremely thermophilic microorganisms of *Sulfolobus*, the only genetically tractable genus of crenarchaea. Sci China Life Sci. 2017; 60(4):370–385. doi: 10.1007/s11427-016-0355-8 28251462 10.1007/s11427-016-0355-8

[29] Li Y, Pan S, Zhang Y, Ren M, Feng M, Peng N, et al Harnessing Type I and Type III CRISPR-Cas systems for genome editing. Nucleic Acids Res. 2016; 44: e34 doi: 10.1093/nar/gkv1044 26467477 10.1093/nar/gkv1044 PMC4770200

[30] Morita R, Nakagawa N, Kuramitsu S, Masui R. An O6-methylguanine-DNA methyltransferase-like protein from *Thermus thermophilus* interacts with a nucleotide excision repair protein. J Biochem. 2008;144(2): 267–77. doi: 10.1093/jb/mvn065 18483064 10.1093/jb/mvn065

[31] Grogan DW. Homologous recombination in *Sulfolobus acidocaldarius*: genetic assays and functional properties. Biochem Soc Trans. 2009; 37(Pt 1): 88–91. doi: 10.1042/BST0370088 19143608 10.1042/BST0370088

[32] Peng N, Deng L, Mei Y, Jiang D, Hu Y, Awayez M, et al A Synthetic Arabinose-Inducible Promoter Confers High Levels of Recombinant Protein Expression in Hyperthermophilic Archaeon Sulfolobus islandicus. Appl Environ Microbiol. 2012; 78(16): 5630–5637. doi: 10.1128/AEM.00855-12 22660711 10.1128/AEM.00855-12 PMC3406144

[33] Napoli A, Zivanovic Y, Bocs C, Buhler C, Rossi M, Forterre P, et al DNA bending, compaction and negative supercoiling by the architectural protein Sso7d of *Sulfolobus solfataricus*. Nucleic Acids Res. 2002; 30(12): 2656–62. 12060682 10.1093/nar/gkf377 PMC117289

[34] Nadal M, Couderc E, Duguet M, Jaxel C. Purification and characterization of reverse gyrase from *Sulfolobus shibatae*. Its proteolytic product appears as an ATP-independent topoisomerase. J Biol Chem. 1994; 269(7): 5255–63. 8106509

[35] Deng L, Zhu H, Chen Z, Liang YX, She Q. Unmarked gene deletion and host-vector system for the hyperthermophilic crenarchaeon *Sulfolobus islandicus*. Extremophiles 2009; 13(4): 735–46. doi: 10.1007/s00792-009-0254-2 19513584 10.1007/s00792-009-0254-2

[36] Contursi P, Jensen S, Aucelli T, Rossi M, Bartolucci S, She Q. Characterization of the *Sulfolobus* host-SSV2 virus interaction. Extremophiles 2006; 10: 615–627. doi: 10.1007/s00792-006-0017-2 16896526 10.1007/s00792-006-0017-2

[37] Guo L, Brügger K, Liu C, Shah SA, Zheng H, Zhu Y, et al Genome analyses of *Icelandic* strains of *Sulfolobus islandicus*, model organisms for genetic and virus-host interaction studies. J Bacteriol. 2011; 193(7):1672–80. doi: 10.1128/JB.01487-10 21278296 10.1128/JB.01487-10 PMC3067641

[38] Peng W, Feng M, Feng X, Liang YX, She Q. An archaeal CRISPR type III-B system exhibiting distinctive RNA targeting features and mediating dual RNA and DNA interference. Nucleic Acids Res. 2015; 43(1):406–17. doi: 10.1093/nar/gku1302 25505143 10.1093/nar/gku1302 PMC4288192

[39] Horton RM, Cai ZL, Ho SN, Pease LR. Gene splicing by overlap extension: tailor-made genes using the polymerase chain reaction. Biotechniques 1990; 8(5):528–35. 2357375

[40] Valenti A, Perugino G, D'Amaro A, Cacace A, Napoli A, Rossi M, et al Dissection of reverse gyrase activities: insight into the evolution of a thermostable molecular machine. Nucleic Acids Res. 2008; 36(14): 4587–97. doi: 10.1093/nar/gkn418 18614606 10.1093/nar/gkn418 PMC2504306

[41] Perugino G, Miggiano R, Serpe M, Vettone A, Valenti A, Lahiri S, et al Structure-function relationships governing activity and stability of a DNA alkylation damage repair thermostable protein. Nucleic Acids Res. 2015; 43(18): 8801–16. doi: 10.1093/nar/gkv774 26227971 10.1093/nar/gkv774 PMC4605297

[42] Miggiano R, Casazza V, Garavaglia S, Ciaramella M, Perugino G, Rizzi M, et al Biochemical and structural studies of the *Mycobacterium tuberculosis O*^*6*^-methylguanine methyltransferase and mutated variants. J Bacteriol. 2013; 195(12): 2728–36. doi: 10.1128/JB.02298-12 23564173 10.1128/JB.02298-12 PMC3697256

