# Supplementary figures and images for: In vivo and in vitro protein imaging in thermophilic archaea by exploiting a novel protein tag
Source: PLoS One. 2017 Oct 3;12(10):e0185791. doi: 10.1371/journal.pone.0185791 (PMC5626487; doi:10.1371/journal.pone.0185791)

## Slide 1
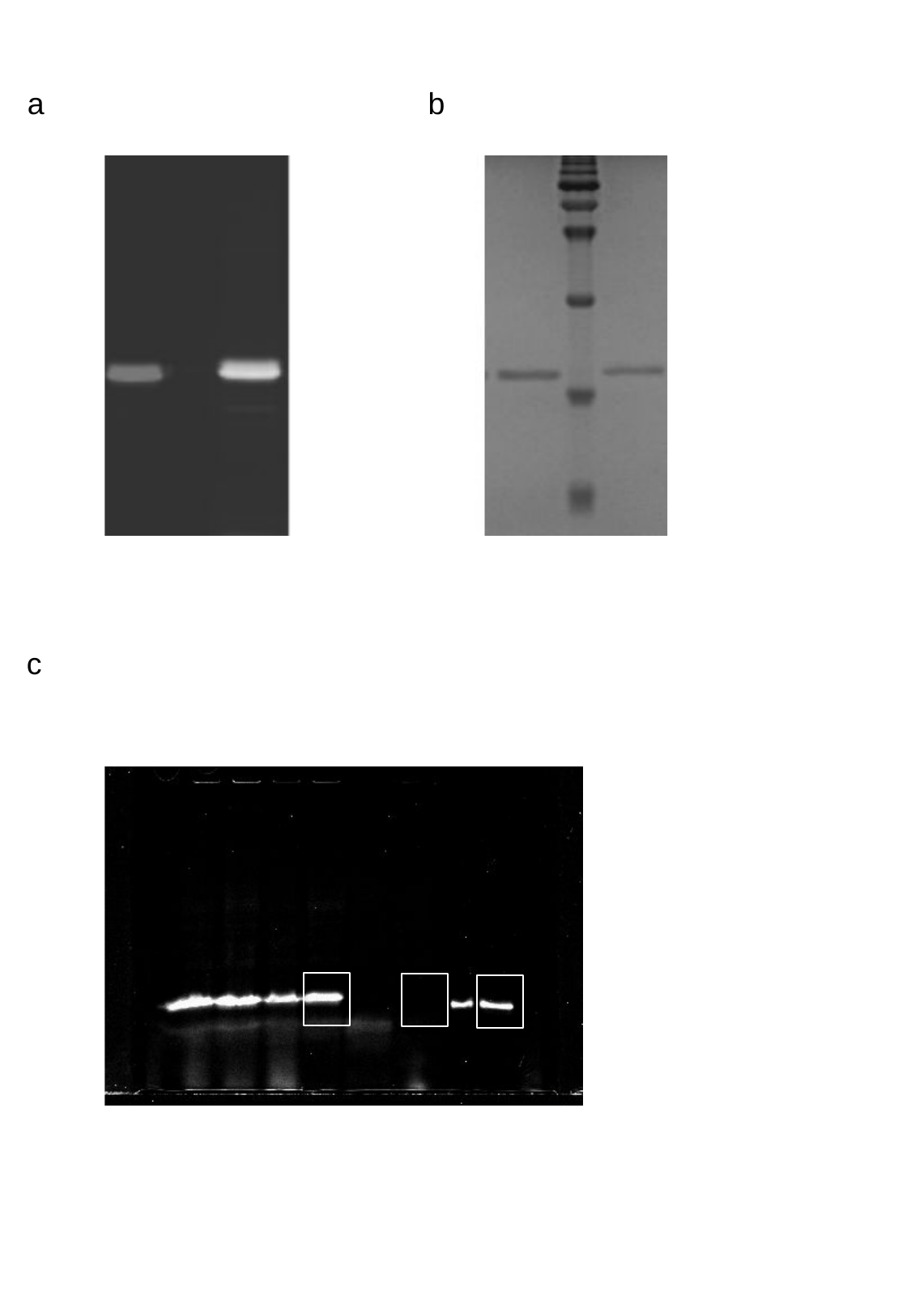

a
b
c

## Slide 2
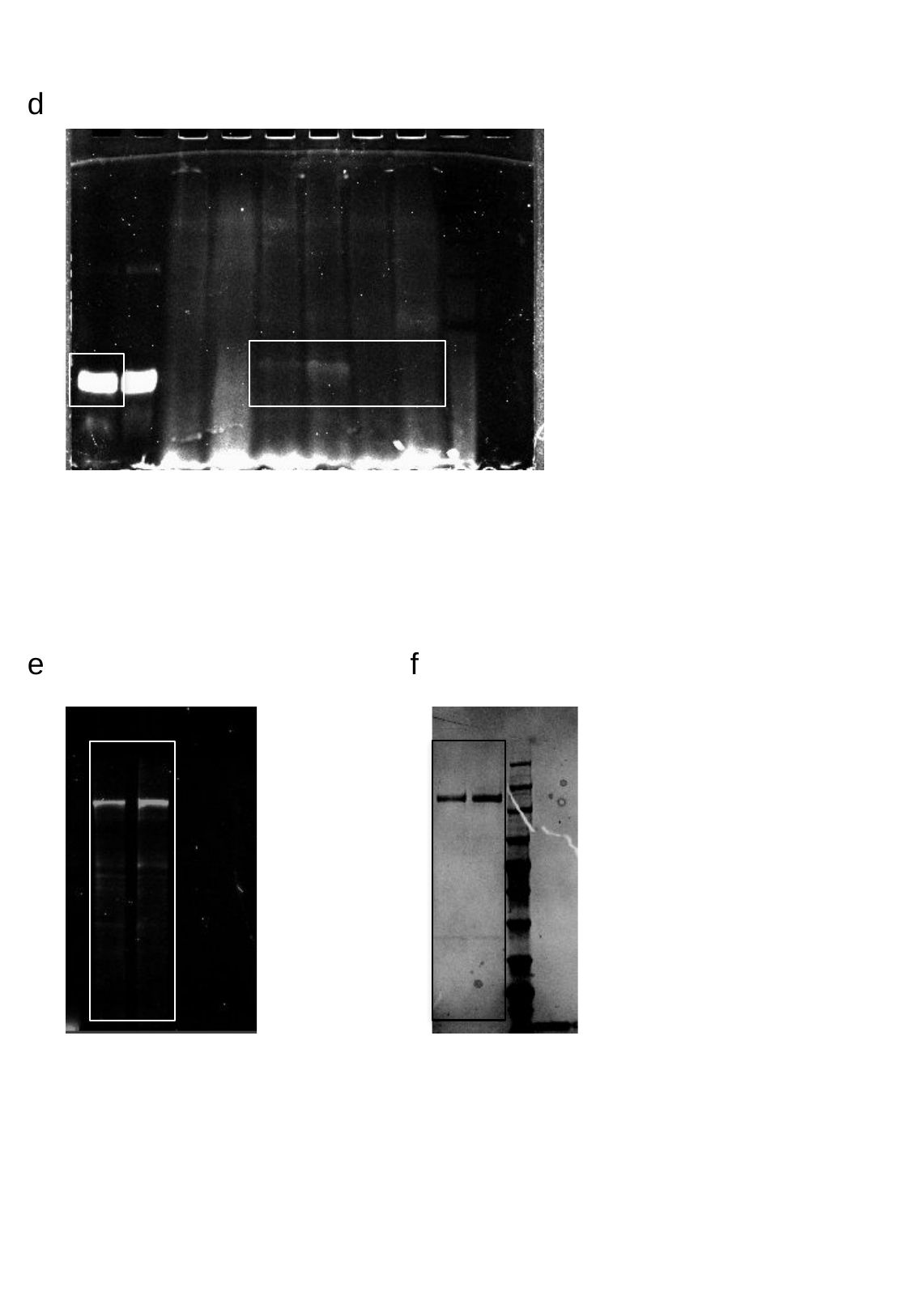

d
e
f

## Slide 3
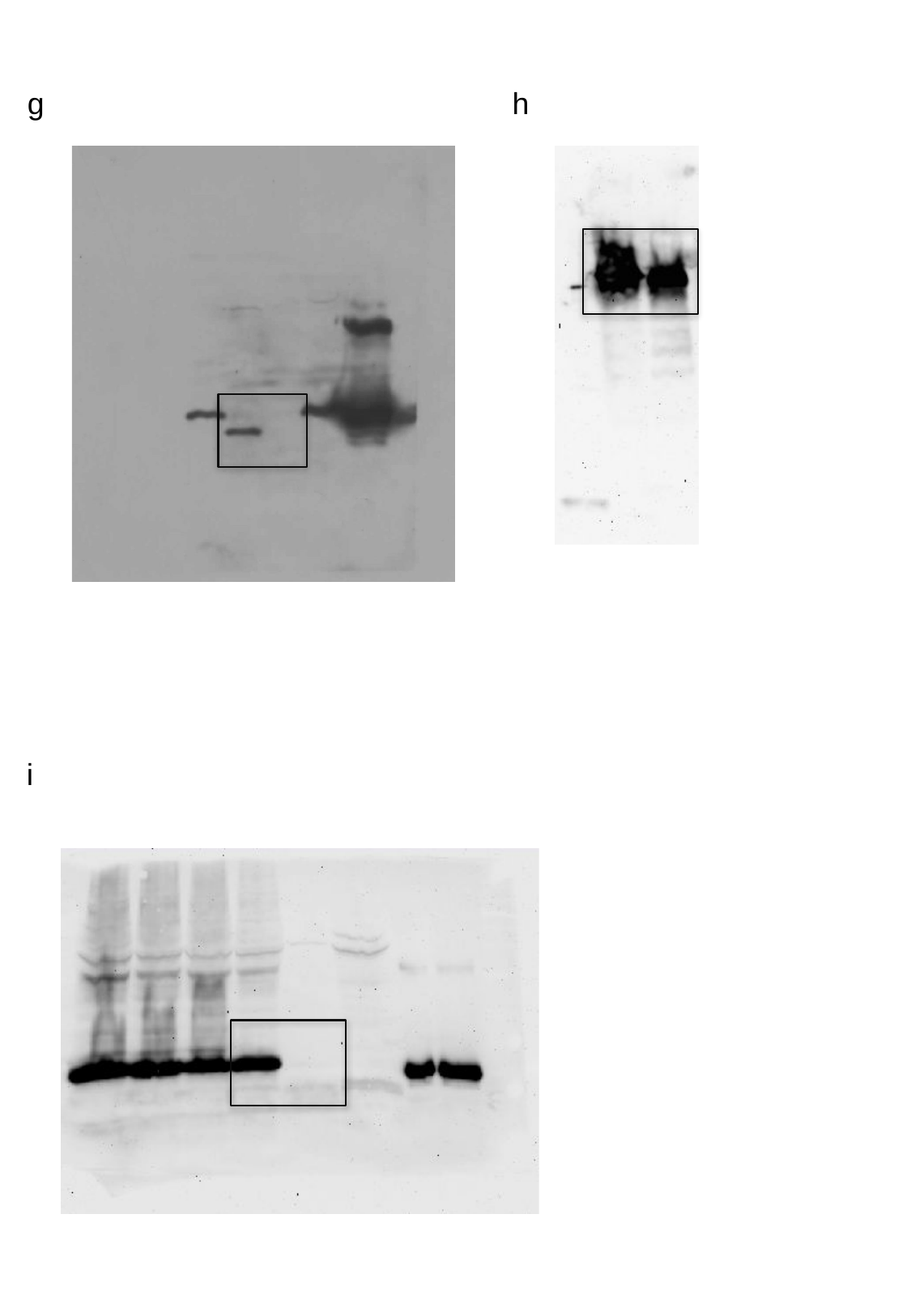

g
h
i

## Slide 4
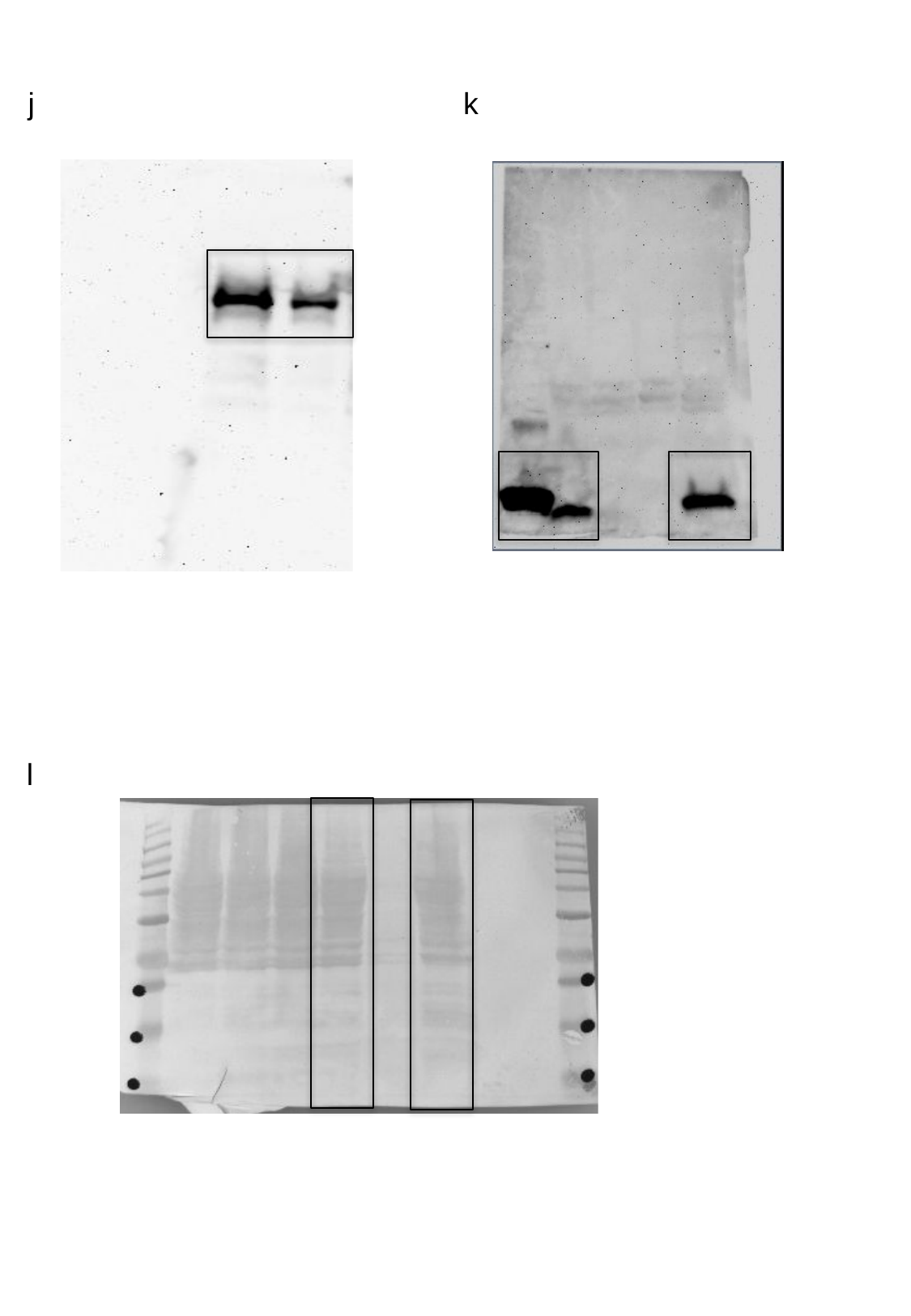

j
k
l

## Slide 5
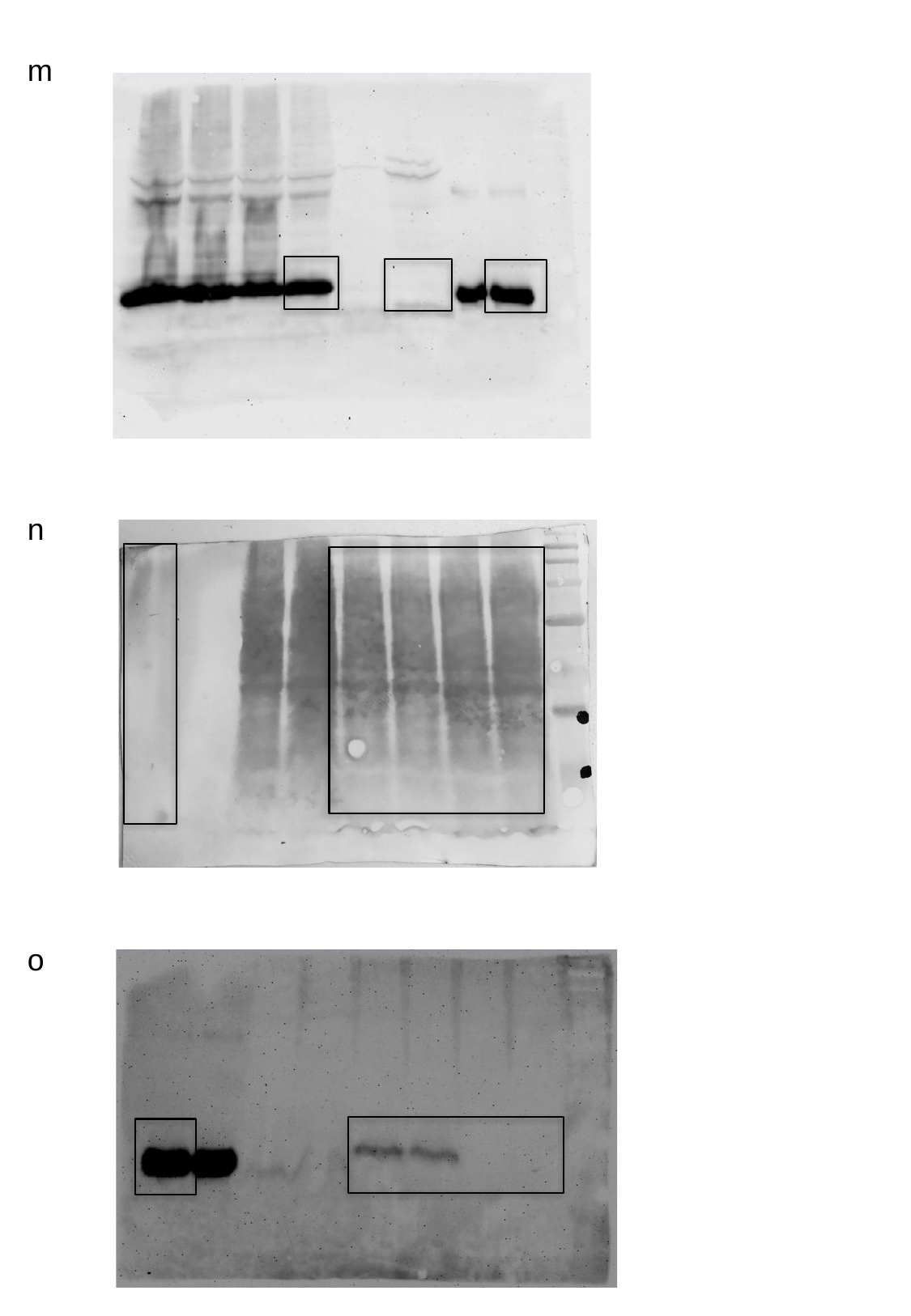

m
n
o

## Slide 6
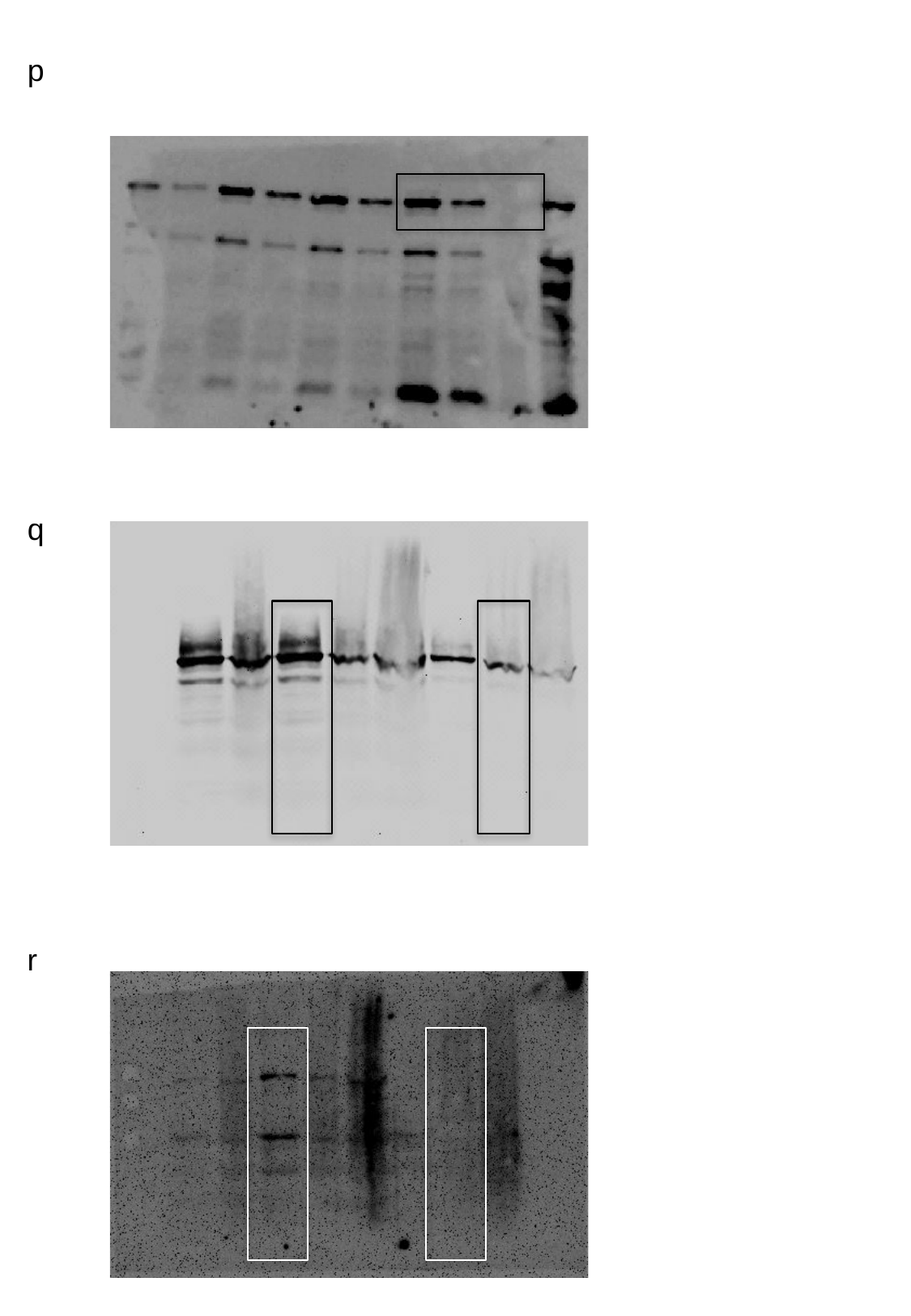

p
q
r

## Slide 7
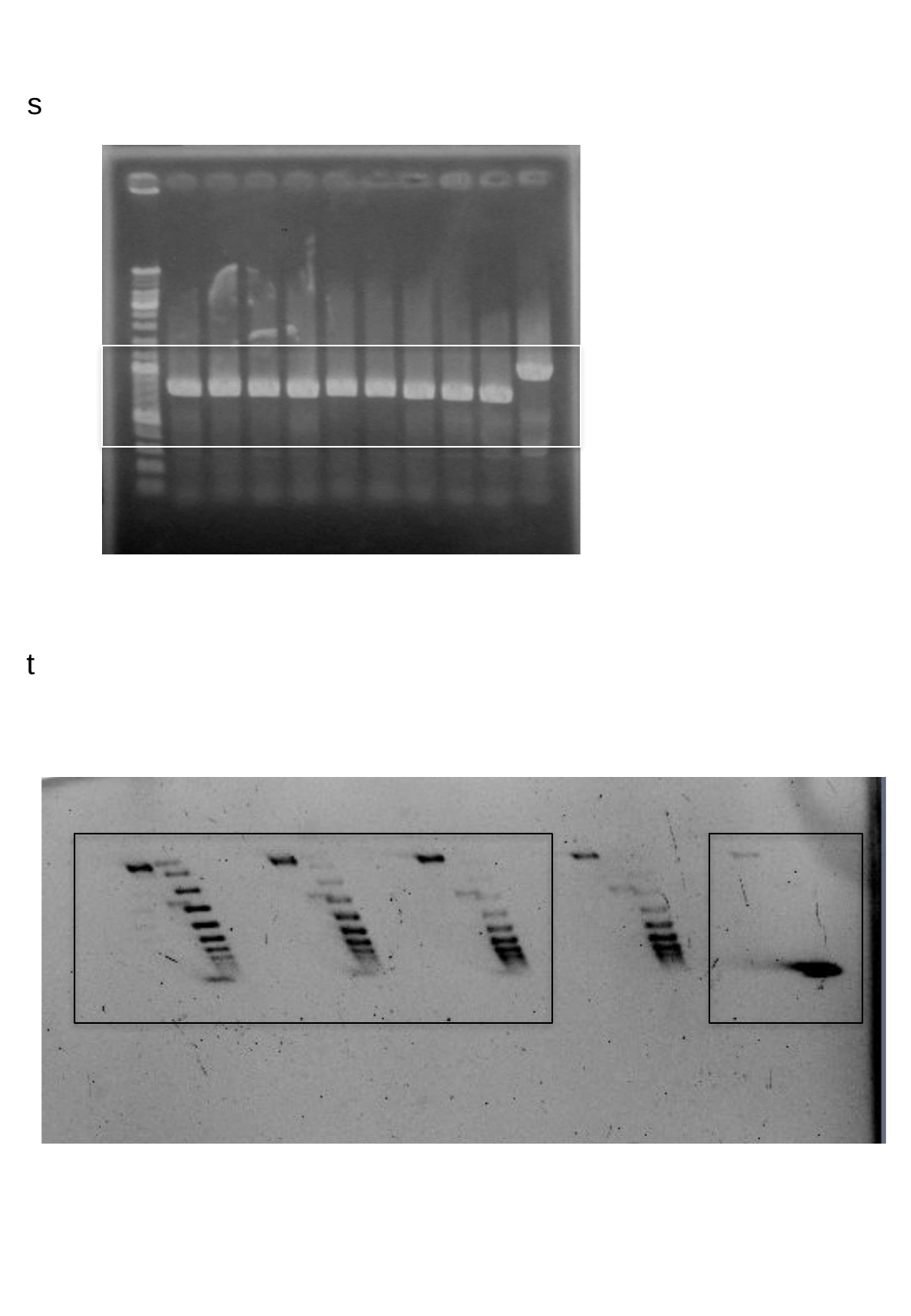

s
t

Supplement: S1 File — (A). Fluorescence imaging of fig. 1B. (B) Coomassie staining of Fig1B. (C). Fluorescence imaging of fig. 4A (top). (D) Fluorescence imaging of fig 4B (top). (E) Fluorescence imaging of fig. 6C. (F) Coomassie staining of Fig. 6C. (G) Western blot Fig 2C (top). (H) Western blot Fig 2C (down) (I) Western blot Fig 3B (top) (J) Western blot of Fig 3B (down) (K) Western blot Fig 3C. (L) Coomassie staining of filter of Fig. 4A (middle) (M) Western blot of Fig 4 A (down) (N) Coomassie staining of filter of Fig 4B (middle) (O) Western blot of Fig. 4B (down) (P) Western blot of Fig 6A (Q) Western blot of Fig. 7A (left) (R) Western blot of Fig.7A (right). (S) Ethidium bromide agarose gel of Fig. 2B. (T) 2D agarose gel after Ethidium bromide staining of Fig 6B. (PPTX) [file pone.0185791.s001.pptx]
